# Albendazole repurposing on VEGFR-2 for possible anticancer application: *In-silico* analysis

**DOI:** 10.1371/journal.pone.0287198

**Published:** 2023-08-16

**Authors:** Nikita Maruti Gaikwad, Pravin Digambar Chaudhari, Karimunnisa Sameer Shaikh, Somdatta Yashwant Chaudhari, Rasha Mohammed Saleem, Mohammad Algahtani, Ahmed E. Altyar, Ghadeer M. Albadrani, Mohamed Kamel, Mohamed M. Abdel-Daim

**Affiliations:** 1 Department of Pharmaceutics, Modern College of Pharmacy, Pune, Maharashtra, India; 2 Department of Pharmaceutical Chemistry, Modern College of Pharmacy, Pune, Maharashtra, India; 3 Department of Laboratory Medicine, Faculty of Applied Medical Sciences, Albaha University, Albaha, Saudi Arabia; 4 Department of Laboratory & Blood Bank, Security Forces Hospital, Mecca, Saudi Arabia; 5 Department of Pharmacy Practice, Faculty of Pharmacy, King Abdulaziz University, Jeddah, Saudi Arabia; 6 Pharmacy Program, Batterjee Medical College, Jeddah, Saudi Arabia; 7 Department of Biology, College of Science, Princess Nourah Bint Abdulrahman University, Riyadh, Saudi Arabia; 8 Department of Pharmaceutical Sciences, Pharmacy Program, Batterjee Medical College, Jeddah, Saudi Arabia; 9 Pharmacology Department, Faculty of Veterinary Medicine, Suez Canal University, Ismailia, Egypt; Gauhati University, INDIA

## Abstract

Drug repurposing is the finding new activity of the existing drug. Recently, Albendazole’s well-known antihelmintic has got the attention of an anticancer drug. Plausible evidence of the interaction of Albendazole with one of the types of tyrosine kinase protein receptor, vascular endothelial growth factor receptor-2 (VEGFR-2) is still not well understood. Inhibition of the VEGFR-2 receptor can prevent tumor growth. The current study investigated the interaction of Albendazole with VEGFR-2.It was found that the said interaction exhibited potent binding energy ΔG = -7.12 kcal/mol, inhibitory concentration (Ki) = 6.04 μM, and as positive control comparison with standard drug (42Q1170A) showed ΔG = -12.35 kcal/mol and Ki = 881 μM. The key residue Asp1046 was formed involved hydrogen bonding with Albendazole. The molecular dynamics simulation study revealed the stable trajectory of the VEGFR-2 receptor with Albendazole bound complex having significant high free energy of binding as calculated from Molecular Mechanics Generalized Born and Surface Area study ΔG = -42.07±2.4 kcal/mol. The binding energy is significantly high for greater stability of the complex. Principal component analysis of molecular docking trajectories exhibited ordered motion at higher modes, implying a high degree of VEGFR-2 and Albendazole complex stability as seen with the standard drug 42Q. Therefore, the current work suggests the role of Albendazole as a potent angiogenesis inhibitor as ascertained by its potential interaction with VEGFR-2. The findings of research will aid in the future development of Albendazole in anticancer therapy.

## Introduction

Cancer is most deadly disease in the world. Cancer treatment is the most difficult task for researchers. As a result, researchers devote time and resources to discovering and developing new therapies for various cancers. Nonetheless, cancer occurrence is anticipated to rise by even more over 50% in the next years, which is regrettable. Ovarian cancer is the seventh leading cause of mortality and morbidity in women globally. There are many types of ovarian cancer, both common and uncommon, such as epithelial ovarian cancer, germ cell tumour, primary peritoneal cancer, fallopian tube cancer, stromal cell tumour, and ovarian cancer [1]. In general, cancer is treated using traditional methods that target the DNA of all cells; however, one important disadvantage of this treatment is that it also affects healthy cells. To address this issue, targeted drug treatment has been established to release a drug to a specific site of receptor while minimizing cell toxicity [2]. To design targeted treatments, scientists must first discover the genetic alterations that allow a cancer cell to mutated. A protein found in cancer cells may become promising approach to target tumor instead of healthy cell [3]. In the treatment of cancer, a broad variety of targeted therapies have been tried. Various drug administration techniques, such as hormone treatments, signal transduction inhibitors, gene expression modulators, apoptosis inducers, angiogenesis inhibitors, immunotherapies, and toxin delivery vehicles, have been shown to have stronger therapeutic action [3]. Angiogenesis inhibitor is one of the successful strategies from this group. Angiogenesis is required for tissue formation, regeneration, and reproduction and is a basic process in the development of a wide range of clinical diseases, including cancer [4]. Angiogenesis, the process by which new blood vessels enter tumour masses, giving oxygen and nutrients to help tumour development and spread, is also important in the transformation of a benign tumour into a malignant one [5]. VEGF has been recognised as an important controller of blood vessel formation and a major facilitator of tumor angiogenesis since its finding in 1983. VEGF is basically falls under growth factor and platelets derived growth factor family that responsible for vasculogenesis and angiogenisis. Overexpression of VEGF in serum revealed an increase in tumour vascular density, which correlates with the degree of malignancy. According to the findings, VEGF levels in ovarian cancer is increased by more than 70% and also in various cancer types. VEGF ordinarily mediates its actions via binding to VEGF receptor present on endothelial cells and by direct acting on VEGFR on tumor cell. Endothelial cell growth and migration can also be aided by VEGF. Endothelial cells are abundant in cancerous tissue [4, 6]. VEGF also controlled number of functions in the body like, invasion, endothelial cell progression, migration, cell permeability and dilation of vessels. So angiogenesis in cancer conditions can be controlled by blocking the tyrosin kinase signalling pathway of VEGF. When VEGF attaches to VEGFR, it causes it to alter conformation, dimerize, and phosphorylate tyrosine [7]. Generally, VEGF acting on both VEGFR1 and VEGFR2 receptor, but more intracellular signalling activation shown by VEGFR2 than VEGFR1. Consequently, VEGFR-2 receptor-associated signalling has emerged as a prospective therapeutic target for cancer therapy [8]. As a result, inhibiting or down-regulating VEGFR-2 signalling is a significant method for creating novel therapeutics for many human malignancies [9]. Drugs that have been licensed or evaluated for other illnesses but demonstrate surprising cytotoxicity against tumor sites, on the other hand, might be repurposed as chemotherapeutic options [10]. Because such drugs are already well-known due to their pharmacokinetics and dynamics features, the odds of failure are quite low, and the development stage becomes highly economical [11]. Furthermore, the majority of antihelmintic drugs that have already been approved are being repurposed as antitumor agents [12, 13]. Notably, fenbendazole, a new tubulin-binding agent with anticancer properties, has recently been proposed as a potential anticancer agent [10]. Mebendazole could also be used for tumor cell inhibition due to its pharmacokinetic features and microtubule depolymerization action [14].

The effectiveness of albendazole as anti-helminthic drug and other benzimidazole class of drug has been related mostly due to its tubulin binding capability, which results in parasite de-polymerization and cell death. Because of their capacity to disrupt microtubule formation, benzimidazoles have been studied for their anti-tumor efficacy in different types of cancer [15]. Albendazole (ABZ) is an approved anthelmintic drug has an anticancer activity and also albendazole shown to have a wide effect on paclitaxel-resistant carcinoma cells [16]. Albendazole demonstrated numerous mechanisms of action in order to show its antihelmintic and anti-cancer potential Albendazole inhibits tubulin polymerization, which is one of the key action pathways. The suppression of tubulin production is directly connected to angiogenesis and vasculogenesis. This binding affects the cell cycle, which leads to cell death by apoptosis. Angiogenesis (the formation of new blood cells) is prevented by inhibiting VEGF receptor overexpression [17].

In this study, mainly focused on efficacy of albendazole as an anticancer agent by studied different pathways. Also studied probability of activity by using PASS prediction, finding showed good activity against carcinogeneic cell. Molecular docking, MD simulations were run to validate stable and converged complexes and learn more about the drug’s thermodynamic characteristics at the specific receptor. The binding energy of ligands to protein molecules determined by MMGBSA (Molecular Mechanics Generalized Born and Surface Area) calculations and it showed significant affinity towards 3VHE target protein. Free surface energy of ABZ bound VEGFR2 receptor analyzed by using PCA (Principal component analysis).

## Materials and methods

### VEGFR-2 protein preparations

The human VEGFR2 kinase domain crystal structure comprises 359 amino acids and a novel pyrrolopyrimidine inhibitor (1-2-fluoro-4-[(5-methyl-5H-pyrrolo[3,2-d]pyrimidin-4-yl)oxy]phenyl-3-[3-(trifluoromethyl)phenyl]urea) 42Q (PDB ID: 3VHE) [18]. It was obtained from the "Protein Data Bank" (https://www.rcsb.org/) and cleaned with the "Biovia Discovery Studio Visualizer" (https://disCover.3ds.com/discovery-studio-visualizer-download). Swiss-pdb viewer 4.1.0 and UCSF Chimera v1.15 were used to optimizing the retrieved protein. Intrinsic water molecules were first removed from the protein’s pocket area and the co-crystallized ligand. Polar hydrogens were inserted, which was proceeded by energy reduction utilizing conjugate gradient and the steepest method. Gasteiger charge potentials were introduced after minimization. All of the protein’s heteroatoms are also eliminated, which may interfere with the receptor’s binding site and cause problems with protein-ligand interaction. The macromolecule’s output structure is then stored in pdbqt format.

### Ligand preparation

**Fig 1:**
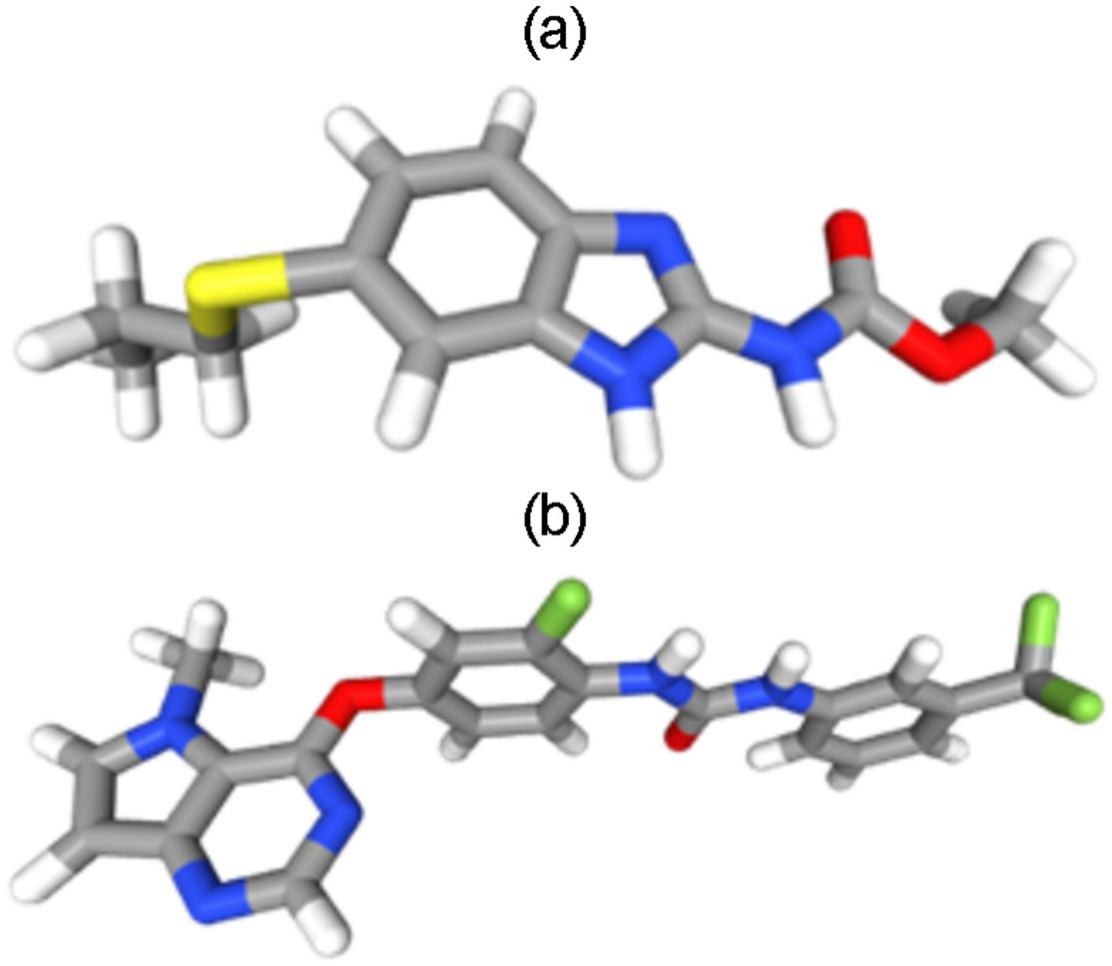
Molecular structures of Albendazole and standard ligand after geometry optimization. (a) Albendazole (ABZ)- (methyl *N*-(6-propylsulfanyl-1*H*-benzimidazol-2-yl)carbamate). (b) 42Q-1-{2-Fluoro-4-[(5-Methyl-5h-Pyrrolo[3,2-D]pyrimidin-4-Yl)oxy]phenyl}-3-[3-(Trifluoromethyl)phenyl]urea.

The structure of ABZ and reference standard 42Q (1-{2-fluoro-4-[(5-methyl-5H-pyrrolo[3,2-d]pyrimidin-4-yl)oxy]phenyl}-3-[3-(trifluoromethyl)phenyl]urea) was downloaded from the public open-access database “PubChem” (https://pubchem.ncbi.nlm.nih.gov/) in 3D-SDF format. The structures of molecules were then translated into PDB format using the "Open Babel GUI" graphic user interface (http://openbabel.org/wiki/MainPage) [19]. AM1 semi-empirical force fields were used to optimize ABZ and 42Q, stored in mol2 file format utilizing the ChemMOP module of the ChemDes webserver (Fig 1).

### Predictions of molecular targets

By entering smiles of desired drugs, the SwissTargetPrediction service was used to investigate specific targets gives an idea about their physiological side effects and cross reactivity for anticancer efficacy. A three—dimensional & two-dimensional similarity index with known ligands is used to determine the probable targets of the provided chemical [20].

### Prediction of biological activity spectra for substances

Prediction of Activity Spectra for Substances (PASS) is a free web service that predicts the biological activities of drug-like chemicals (http://www.way2drug.com/passonline). Based on multilevel neighbourhoods of atom (MNA) descriptors and a modified Bayesian algorithm containing more than 300,000 organic compounds with more than 6,825 distinct biological activities, it also predicts a structure-activity relationship (SAR). Thus, PASS may be applied to improve and well-target chemical production and biological testing. The anticancer activity of the ABZ and standard drug 42Q was anticipated, and the findings are shown in Table 1. A substance’s estimated activity is forecasted as probable activity (Pa) and probable inactivity (Pi). Only compounds with a Pa greater than Pi are considered suitable for a certain therapeutic activity [21].

**Table 1:** PASS prediction coefficients of ABZ and 42Q against tumor cell lines and other protein receptors related to cancer progression.

Activity	ABZ		42Q	
	Pa	Pi	Pa	Pi
**Antineoplastic (melanoma)**	0,348	0,015	0,302	0,076
**Antineoplastic (solid tumors)**	0,320	0,075	0,348	0,059
**Antineoplastic (lymphocytic leukemia)**	0,248	0,026		
**Antineoplastic (endocrine cancer)**	0,212	0,039	0,161	0,106
**Protein kinase inhibitor**	0,199	0,015	0,438	0,023
**Platelet derived growth factor receptor kinase inhibitor**	0,323	0,137	0,665	0,009
**MAP3K5 inhibitor**	0,361	0,048		
**Transcription factor STAT inhibitor**	0,344	0,064		
**Angiogenesis inhibitor**	0,307	0,068	0,848	0,004
**Anticarcinogenic**	0,221	0,100		
**VEGF antagonist**			0,780	0,004
**VEGFR-2 antagonist**			0,730	0,004
**TIE antagonist**			0,743	0,003
**TIE-2 antagonist**			0,740	0,003

### Molecular docking

This research aims to shed insight on the binding properties of 3VHE protein using geometrically optimized chemicals like ABZ and standard 42Q. Before being employed in molecular interaction studies, proteins were checked for missing side-chain residues using the open Molecular Mechanics (MM) simulation tool (https://openmm.org/). Autodock v 4.2.6 was used for molecular docking investigations. The binding cavity was established using the co-crystallized X-ray structure of VEGFR-2 protein from the RCSB PDB. Within three spaces of the co-crystallized ligand, the residue locations were computed. The energy was minimized using the steepest descent and conjugate gradient techniques after cavity selection. Finally, the receptor and target molecules were stored in pdbqt format after combining the nonpolar hydrogens. Grid boxes with a spacing of 0.3 were made. To get the minimum free energy of binding (G), docking studies of the protein-ligand complex were performed utilizing the Lamarckian Genetic Algorithm (LGA). Three replicates of molecular modelling experiments were carried out, with default parameters like number of solutions (50 in each), number of evaluations (2500000), maximum number of generations (2700) etc. After docking, the Root Mean Square Deviation clustering maps were created by re-clustering with clustering tolerances of 0.25, 0.5, and 1 in order to obtain the best cluster with the lowest energy score and a high number of populations, respectively.

### Molecular dynamics simulation

The Desmond software Vs 2020.1 from Schrödinger was used to do MD simulations on docked complexes for ABZ and 42Q with protein (PDB ID: 3VHE). Triplicate sampling was performed with the same condition for each MD run to achieve better results. This system employed the OPLS-2005 force field [22–24] and an explicit solvent model with simple point charger (SPC) water molecules [25]. To neutralise the charge, Na+ ions were added. NaCl solution (0.15M) was used to simulate the physiological environment. To retrain over the protein-ligand complex, the system was first equilibrated using NVT ensemble for 200 ps. This system was then exposed to a 12 ps run for equilibration and minimization using the NPT ensemble. The NPT ensemble was built using the Nose-Hoover chain coupling method [26]. All simulations were performed at a temperature of 27°C, with a relaxation time of 1.0 ps and a pressure of 1 bar. A time step of 2 fs was chosen. For pressure regulation, the Martyna-Tuckerman–Klein chain coupling scheme [27] barostat technique with a relaxation duration of 2 ps was utilised. The particle mesh Ewald method [28] was used to determine long-range electrostatic interactions, with the Coulomb interaction radius set at 9 cm. For each trajectory, the bonded forces were estimated using the RESPA integrator with a time step of 2 fs. To measure the stability of MD simulations, the root mean square deviation (RMSD), radius of gyration (Rg), root mean square fluctuation (RMSF), solvent accessible surface area (SASA), and a number of hydrogen bonds were computed. RMSD clustering of individual trajectories for 200 ns of 3VHE+apo, 3VHE+ABZ and 3VHE+42Q were carried out using Desmond trajectory clustering module. The RMSD matrix was built by considering frequency of 20 and possible clusters 10. Initially the highest populated cluster from all the three apo. ABZ and 42Q bound proteins (3VHE) and compared them by superimposing to investigate the conformational changes. Then top two highest and second highest populated clusters are compared for conformation variation at binding cavities of the ligand.

### MMGBSA calculations

Molecular Mechanics Generalized Born surface area (MM-GBSA) was used to calculate the binding free energy (ΔG_bind_) of docked complexes during MD simulations of 3VHE complexed with ABZ and 42Q. (Schrodinger suite, LLC, New York, NY, 2017–4). The binding free energy was estimated using the OPLS 2005 force field, the VSGB solvent model, and rotamer search techniques. The MD trajectories frames were chosen at 10 ns intervals after the MD run. Eq 1 was used to compute the total free energy binding:

$$\Delta {\text{G}}_{\text{bind}}={\text{G}}_{\text{complex}}-\left({\text{G}}_{\text{protein}}+{\text{G}}_{\text{ligand}}\right)$$ (1)

Where,

ΔG_bind_ = binding free energy,

G_complex_ = free energy of the complex,

G_protein_ = free energy of the target protein, and

G_ligand_ = free energy of the ligand.

The MMGBSA outcome trajectories were analyzed further for post dynamics structure modifications.

### Principal component analysis (PCA)

During a 200 ns simulation of the human VEGFR2 kinase domain (PDB ID: 3VHE) complexed with ABZ, PCA analysis was used to recover the global movements of the trajectories. A covariance matrix was generated as described to calculate the PCA. Conformational analysis of Albendazole in-bound complex was done by using 10 appropriate modes of conformation. The main component was calculated as trajectories, and a comparison of the first highest mode (PC1) versus the second highest mode (PC2), followed by PC2 and PC3, and finally, PC9 and PC10 modes were investigated. Geo measures v0.8 were used to calculate the principal components based on eigenvectors from the correlation matrix on an ABZ-bound complex. The MD trajectory versus principal components of trajectory motion was recorded in a 2D plot using the Matplotlib python package using Geo measures, including a comprehensive library of g_sham [29].

## Results and discussion

### Molecular target predictions

**Fig 2:**
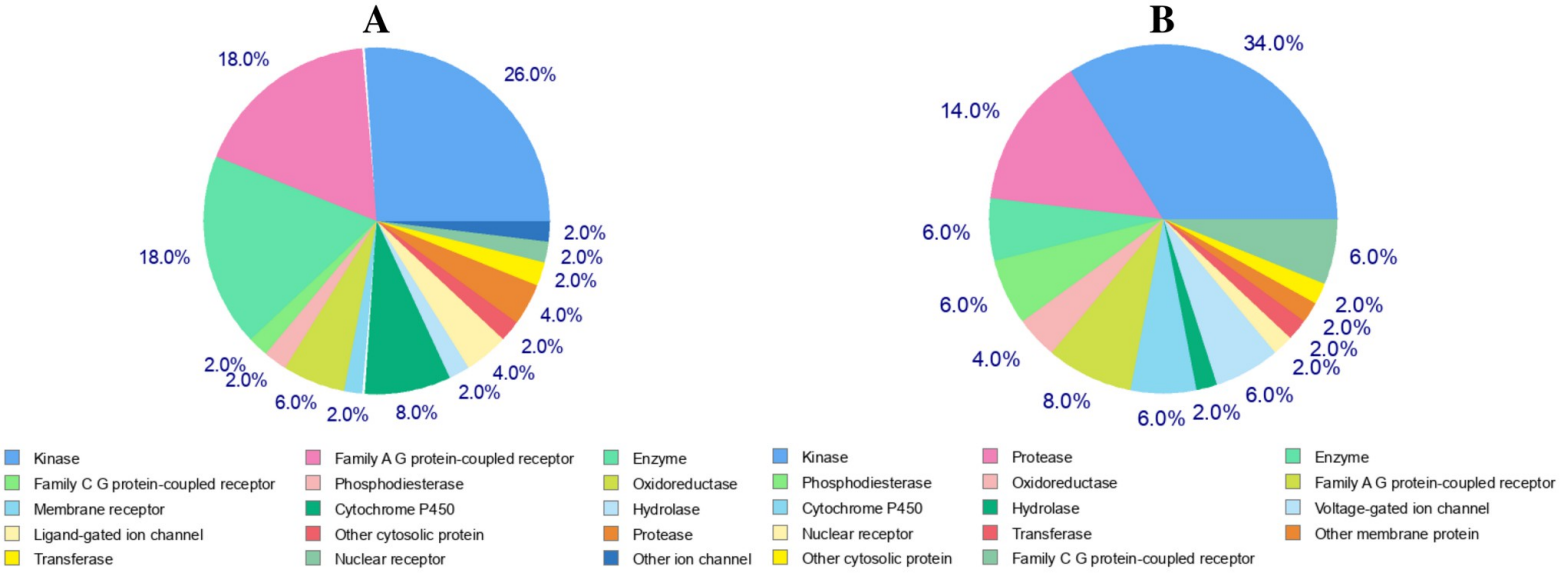
Pie-chart of top-50 of target predicted for selected molecules (A: ABZ, B: 42Q).

Molecular target studies were projected based on their similarities to known compounds to estimate their targets to conduct the molecular mechanisms behind a certain phenotypic or bioactivity and rationalize any side effects. The top 50 results of the closely related receptors in 2D/3D were shown as a pie chart based on Target, Popular Name, Uniprot ID, ChEMBL-ID, Target Class, Probability, and Reported actives (Fig 2). As can be seen, ABZ predicts 26% Kinase receptor, 18% family α G protein-coupled receptor, and 18% Enzymes while 42Q has 34% Kinases and 14% protease, and 6% Enzymes.

Overall, the findings demonstrated the efficacy of ABZ as an anticancer treatment that targets the MAP kinase p38 alpha, protein-tyrosine kinase erbB-2, nuclear factor-kappa kinase beta subunit, Serine/threonine-protein kinase, Tyrosine-protein kinase JAK3, Tyrosine-protein kinase JAK2, VEGFR-1.

### Prediction of activity spectra for substances (PASS) prediction

PASS employed both ABZ and 42Q to forecast the biological spectrum. Out of a maximum likelihood score of 1, it calculates the probability of activity and inactivity against cancer and non-cancerous cells. With active coefficients of 0.348, 0.320, 0.248, and 0.212, ABZ showed considerable anticarcinogenic action against melanoma, solid tumors, lymphocytic leukemia, and endocrine cancer in the PASS filter (Table 1). 42Q was shown to be effective against melanoma (0.302) and solid tumors (0.348). Nonetheless, both ABZ and 42Q displayed significant activity on endocrine cancer; Platelet-derived growth factor receptor kinase, MAP3K5, Transcription factor STAT, Angiogenesis, etc. These findings point to a high likelihood of anticarcinogenic action. This bioactivity investigation confirms albendazole’s importance in protecting human health against tumor development and inflammation.

### Molecular docking

**Fig 3:**
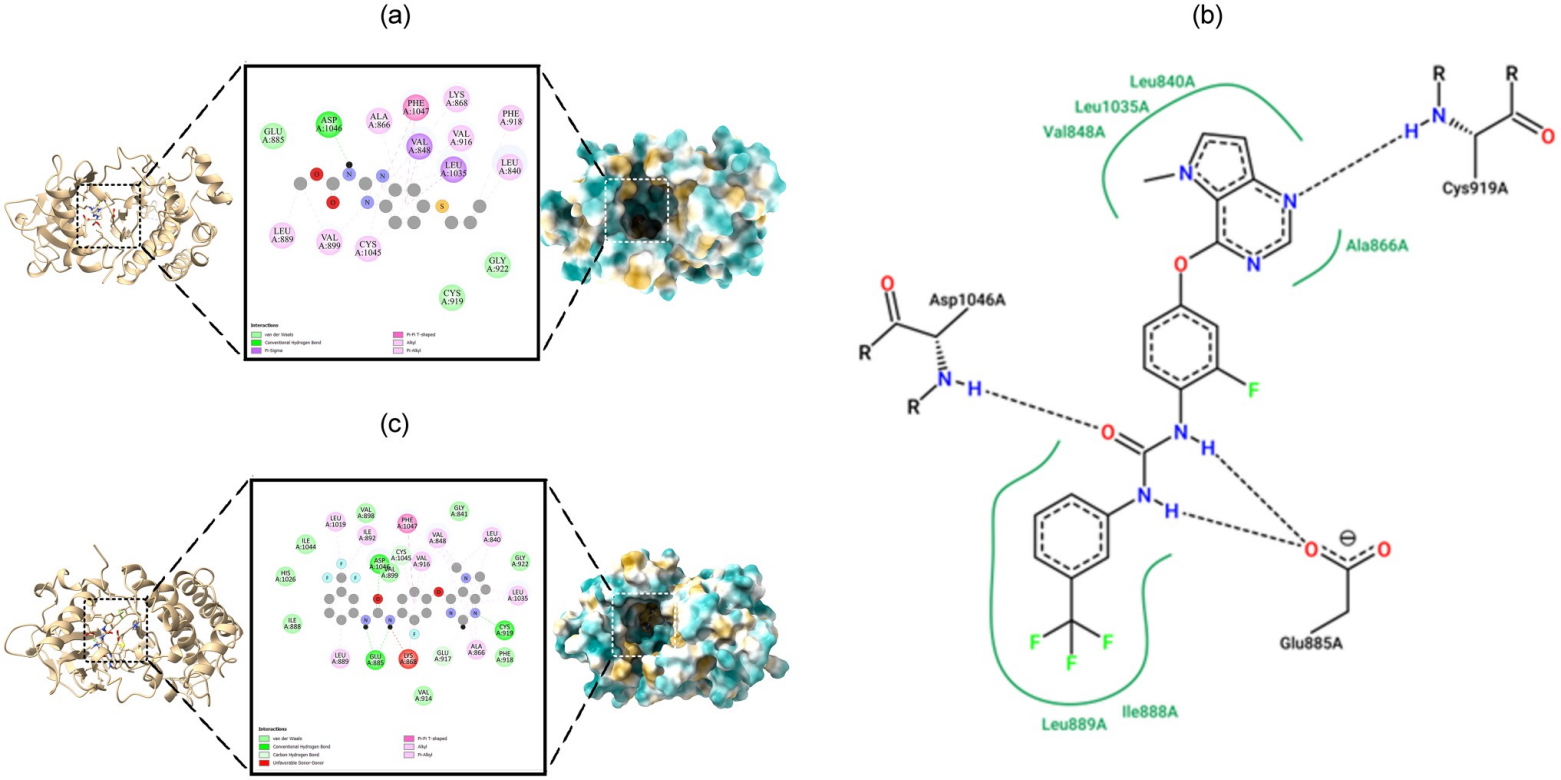
Binding pose of molecular docking. (A) 3VHE+ABZ. A left panel displays the ribbon structure of 3VHE associated with ABZ in the stick model. A middle panel displays the 2D interaction of ABZ (disc-shaped) surrounding the binding cavity residues in 3VHE. A right panel displays the surface view of the 3VHE displaying binding cavity and accommodation of ABZ. (B) A left panel displays ABZ 2D interaction at the binding cavity in the wireframe. A right panel displays 42Q 2D interaction at the binding cavity in the wireframe. (C) 3VHE+42Q. A left panel displays the ribbon structure of 3VHE associated with 42Q in the stick model. A middle panel displays the 2D interaction of 42Q (disc-shaped) surrounding the binding cavity residues in 3VHE. A right panel displays the surface view of the 3VHE displaying binding cavity and accommodation of 42Q.

All the binding energy scores are calculated from the best cluster (95%) that falls within the lowest RMSD 0.25 Å. With the lowest binding energy, (G– 7.12 kcal/mol) and inhibitory concentration, Ki (6.04 M), ABZ showed a considerable binding affinity for the human VEGFR2 kinase domain (PDB ID: 3VHE). During the interaction of the ligand ABZ, Asp1046 residues at the binding cavity of the protein were involved in conventional hydrogen bonding. Apart from hydrogen bonding, van der Waal’s interactions by amino acid residues were also involved in non-bonded interaction with the ligand (Fig 3A and 3B left panel). While the standard ligand 42Q displayed a high binding affinity for 3VHE protein where free energy binds were recorded as -12.35 kcal/mol and inhibitory concentration (Ki) 881 pM, respectively. Glu885, Cys919, Asp1044 residues in the binding cavity of3VHE formed conventional hydrogen bonds (Fig 3B, right panel and 3C).

### Molecular dynamics and simulation

**Fig 4:**
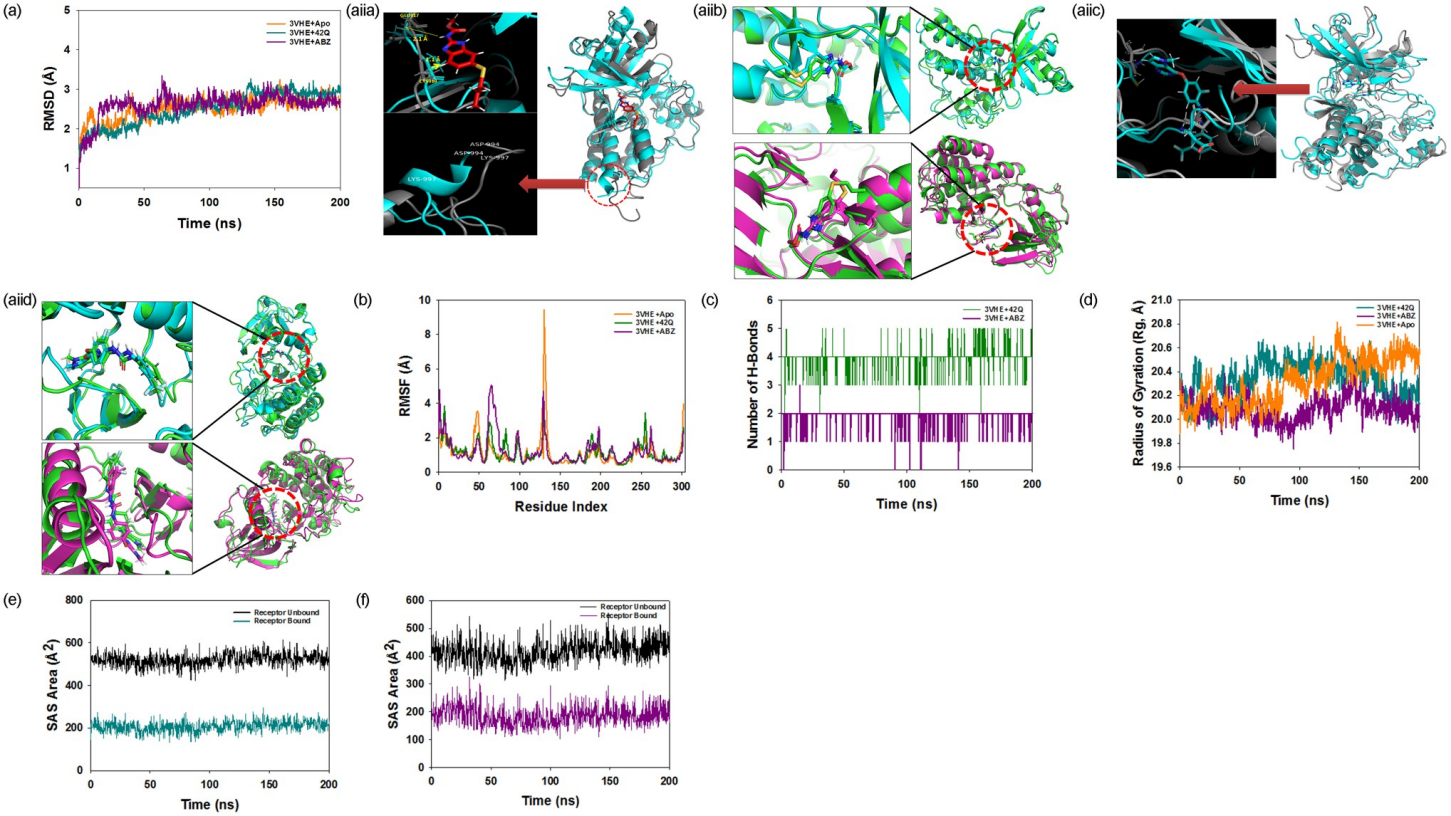
Analysis of non parametric clustering of RMSD for ABZ and 42Q and MD simulation trajectories of 200 ns time scale. [A(i)] RMSD plot displaying the molecular vibration of Cα backbone of 3VHE-apo (orange), 3VHE+ABZ (dark pink), and 3VHE+42Q (dark green). A(ii) a. Comparison of conformations Apo and ABZ bound top RMSD cluster. A (ii) b. Comparison of conformations ABZ bound top thee RMSD clusters. A (ii) d. Comparison of conformations 42Q bound top three RMSD clusters. A (ii) c. Comparison of conformations Apo and 42Q bound top RMSD cluster. [A (ii)] Non parametric clustering of RMSD for ABZ and 42Q and conformational comparison with apo-protein highest populated cluster as well as intra top three highest populated clusters. B: RMSF plots show the fluctuations of respective amino acids throughout the simulation time 200 ns for -apo (orange), 3VHE+ABZ (dark pink), and 3VHE+42Q (dark green). (C) Number of hydrogen bonds formed between -apo (orange), 3VHE+ABZ (dark pink), and 3VHE+42Q (dark green) during 200 ns simulation time scale. (D) The radius of gyration plots for the deduction of compactness of protein -apo (orange), 3VHE+ABZ (dark pink), and 3VHE+42Q (dark green). Solvent accessible surface area (SAS Area) displaying the ligand-bound and unbound area (Black) at the binding pocket. (E) 3VHE+ABZ (dark green). (F) 3VHE+42Q (dark green).

The stability and convergence of the 3VHE+ABZ and 3VHE+42Q complexes were investigated using molecular dynamics and simulation (MD). When comparing the root mean square deviation (RMSD) data, a simulation of 200 ns revealed stable conformation. The RMSD of Cα-backbone of 3VHE bound to ABZ exhibited a deviation of 0.2 Å (Fig 4A (i)), while 42Q displayed a deviation of 0.64 Å. However, 3VHE protein without ligand (3VHE-Apo) exhibited a deviation from the beginning to the end of simulation 0.63 Å. Similar RMSD of ligand bound protein and apo-protein suggested least perturbations and stable conformations. Therefore, it can be suggested that ABZ and 42Q bound 3VHE is quite stable in complex due to the higher affinity of the ligand. Comparison of RMSD clustering having highest population of Apo protein and ABZ bound to 3VHE proteins displayed little conformational changes (Fig 4A (ii)). At the binding cavity of ligand ABZ, couple of residue forming conventional hydrogen bonds viz. Glu917 and Cys919 where we have observed Glu917 pushed away (distance 2.1 Å) from the original position in ligand bound state while comparing with the position in apo protein where the orientation of the residue is much straighter (Fig 4A (ii), left panel, up). RMSD of the deviated residue of ligand bound state and apo state was measured to be RMSD 0.23 Å. The other residue Cys919 is pulled toward ligand ABZ(distance 1.1 Å) while in the apo protein Cys919 seemed to be straighter and unaltered (RMSD 0.21 Å) (Fig 4A (ii), left panel, up). On the other hand, at the C-terminal end residues Asp994-Lys997 of the apo protein conformed randomly into a loop structure whereas, in ABZ bound 3VHE protein a partial helix formed at that position (RMSD 1.5Å) (Fig 4A (ii), left panel, down). Formation of helix structure made the C-terminal of 3VHE more stable than the loop orientation. The top three clusters having highest populations are also compared to see the positional variances of the ligand. Comparison of conformation of top two cluster 1 and cluster 2 displayed aligned ligands having small positional deviation (RMSD 0.25Å) (Fig 4A (ii), right panel, green-cyan, up). Whereas, comparison of cluster 1 and cluster 3 showed significant deviation of ligand twisted around the axis (Fig 4A (ii), right panel, green-magenta, down). Therefore, conformational stability is achieved in the untwisted form of the ligand at the binding cavity than the twisted conformation. Apo and 42Q bound top most clusters having highest population showed significant movements of residues to achieve high stability. In 42Q bound 3VHE (Fig 4A (ii), cyan) two key residues Cys919 and Asp1046 moved apart to form spatial arrangement with the ligand for hydrogen bond formation. While Glu885 pulled toward the ligand to attain a stable hydrogen bond formation where in apo protein the residue lie distantly apart (Fig 4A (ii)). Comparative analysis of top two cluster 1 and cluster 2 having highest population of 42Q bound 3VHE trajectories displayed least ligand mobility from its axis exhibiting very stable complexes (Fig 4A(ii)). The plots for root mean square fluctuations (RMSF) displayed small spikes of fluctuation except at amino acid residue 60 (4.5 Å) and 130–140 (8 Å) in 3VHE-ABZ complex, while the rest of the residues less fluctuated during the entire 200 ns simulation (Fig 4B, dark pink) indicating the stable amino acid conformations during the simulation time. The high fluctuations in ABZ bound protein residues due to loop conformation. However, in 42Q bound protein showed significant fluctuation at 130–140 residues (4.3 Å) (Fig 1B, dark green). In contrast, RMSF values of the 3VHE-apo displayed significant fluctuations, and at 130–140 residues, the fluctuation was found to be 9 Å, signifying very flexible unstable residues ordered into a loop conformation (Fig 4B, orange). Therefore, from RMSF plots, it can be suggested that the structures of 3VHE were stable during simulation in ABZ and 42Q bound conformations. The number of hydrogen bonds between protein and ligand suggests the significant interaction and stability of the complex. A significant number of hydrogen bonds showed between 3VHE & ABZ and 42Q throughout the simulation time 200 ns (Fig 4C). Consistent numbers of hydrogen bonds are observed between 3VHE and ABZ (Average 2 numbers) and 42Q (Average 4 numbers) that might facilitate to conform into stable complex (Fig 4C). The gyration radius is a measurement of the protein’s compactness. Here in this study, 3VHE Cα-backbone bound to ABZ displayed lowering of radius of gyration (Rg) from 20.3 Å to 20.0 Å (Fig 4D, dark pink), and 3VHE Cα-backbone bound to 4Q2 displayed stable as well as lowering of peaks from 20.2 to 20.0 Å (Fig 4D). Similarly, 42Q bound protein displayed the lowering of Rg from 20.3 to 20.2 Å (Fig 4D). Lowering of RMSD indicates the compactness of protein ligand complex. On the other hand, apoprotein (3VHE-apo) displayed significant uneven fluctuations and was devoid of stability, indicating the less compact orientation of the protein (Fig 4D, orange). After Rg analysis, similar patterns were identified in solvent accessible surface area (SASA) in both ligand-bound and unbound states. The unbound state of ligand 3VHE revealed a large surface area accessible to solvent (Fig 4E and 4F, black), but binding with 42Q and ABZ resulted in a lower SASA value than the unbound state (Fig 4E and 4F, dark green and dark pink, respectively). The ligand ABZ and 42Q binding causes the proteins to become more compact and less flexible.

### Molecular mechanics generalized born and surface area (MMGBSA) calculations

The MMGBSA technique is widely used to determine the binding energy of ligands to protein molecules. The binding free energy of each of the 3VHE+ABZ and 3VHE+42Q complexes was calculated and the involvement of additional non-bonded interaction energies. The average binding energy of the ABZ bound to 3VHE was found to be -42.07 ± 2.4 kcal/mol, whereas the average binding energy of 42Q was determined to be -80.94 ± 2.25 kcal/mol (Table 2). G_bindCoulomb_, G_bindCovalent_, G_bindHbond_, G_bindLipo_, G_bindSolvation_, and G_bindvdW_ interactions are all examples of non-bonded interactions that affect G_bind_. G_bindvdW_, G_bindLipo_, G_bindHbond_, and G_bindCoulomb_ energies contributed the most to obtain the average binding energy across all types of interactions. The G_bindSolvation_ and G_bindcovalent_ energies, on the other hand, contributed the least to the final average binding energies.

**Table 2:** Non-bonded interactions between ligand molecules and the target protein contribute binding energy to MMGBSA.

Energies	ABZ (kcal/mol)*	42Q (kcal/mol)*
**dG Bind**	-42.07±2.4	-80.94±2.25
**dG Coulomb**	-8.68±2.44	-21.39±1.51
**dG Hydrogen Bond**	-2.03±0.77	-2.39±0.18
**dG Solvation**	12.4 = 21±2.21	22.48±0.67
**dG Covalent**	2.01±0.72	2.92±0.312
**dGLipo**	-8.15±0.09	-21.47±0.81
**dGvdW**	-35.35±6.44	-59.30±5.27

*Values represented in mean±SD

**Fig 5:**
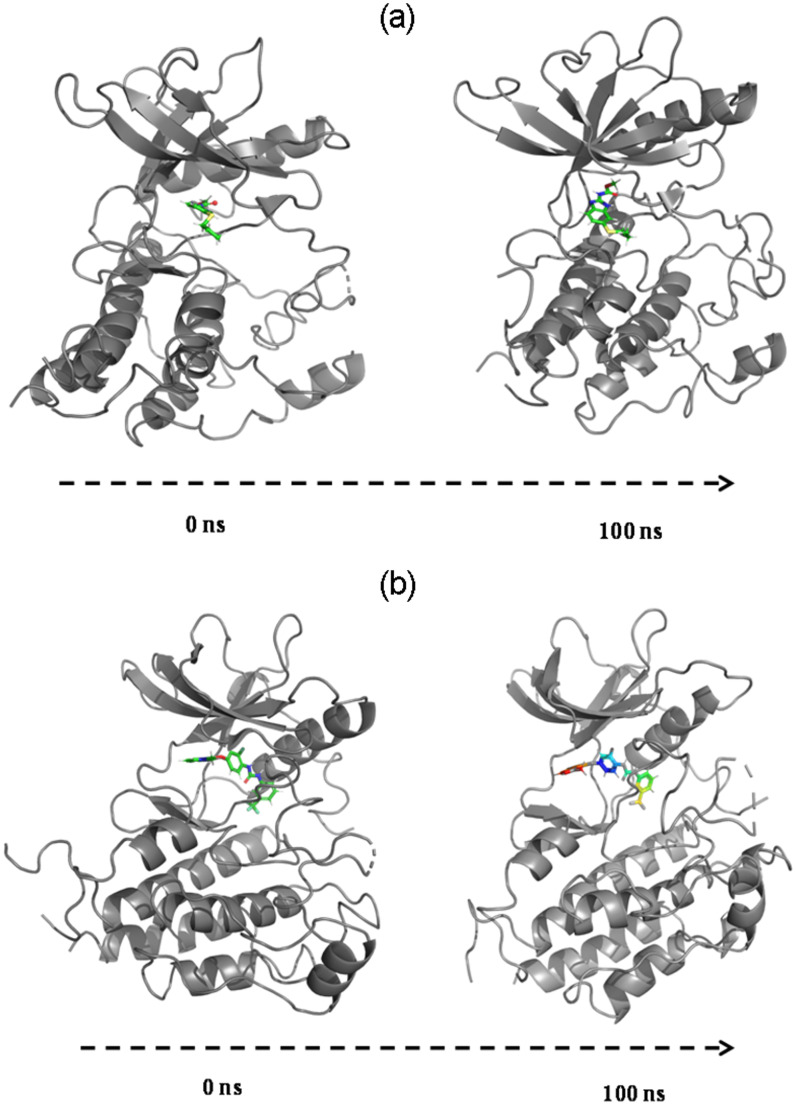
Positional changes comparison of first (0 ns) and last frame (200 ns) of ligand (A) ABZ and (B) 42Q bound to 3VHE from MMGBSA trajectories.

MMGBSA trajectories of 3VHE+ABZ and 3VHE+42Q complexes are further analyzed for positional variances of the respective ligand at the binding pocket. Complex 3VHE with ABZ displayed change in orientation in the ligand position from 0 to 200 ns (Fig 5A). While least arrangement is observed in the 42Q ligand (Fig 5B). MMGBSA trajectory of ABZ and 42Q with 3VHE displayed a very stable configuration throughout the simulation. Moreover, high binding energies were observed due to stable conformation with the protein-ligand complex. Therefore, for the entire simulation studies and MMGBSA analysis, it can be suggested that both ABZ and 42Q have significant affinity for the target 3VHE and can be potential inhibitors of drug molecules.

### Analysis of principal component (PCA) with free energy surface/landscape (FEL)

**Fig 6:**
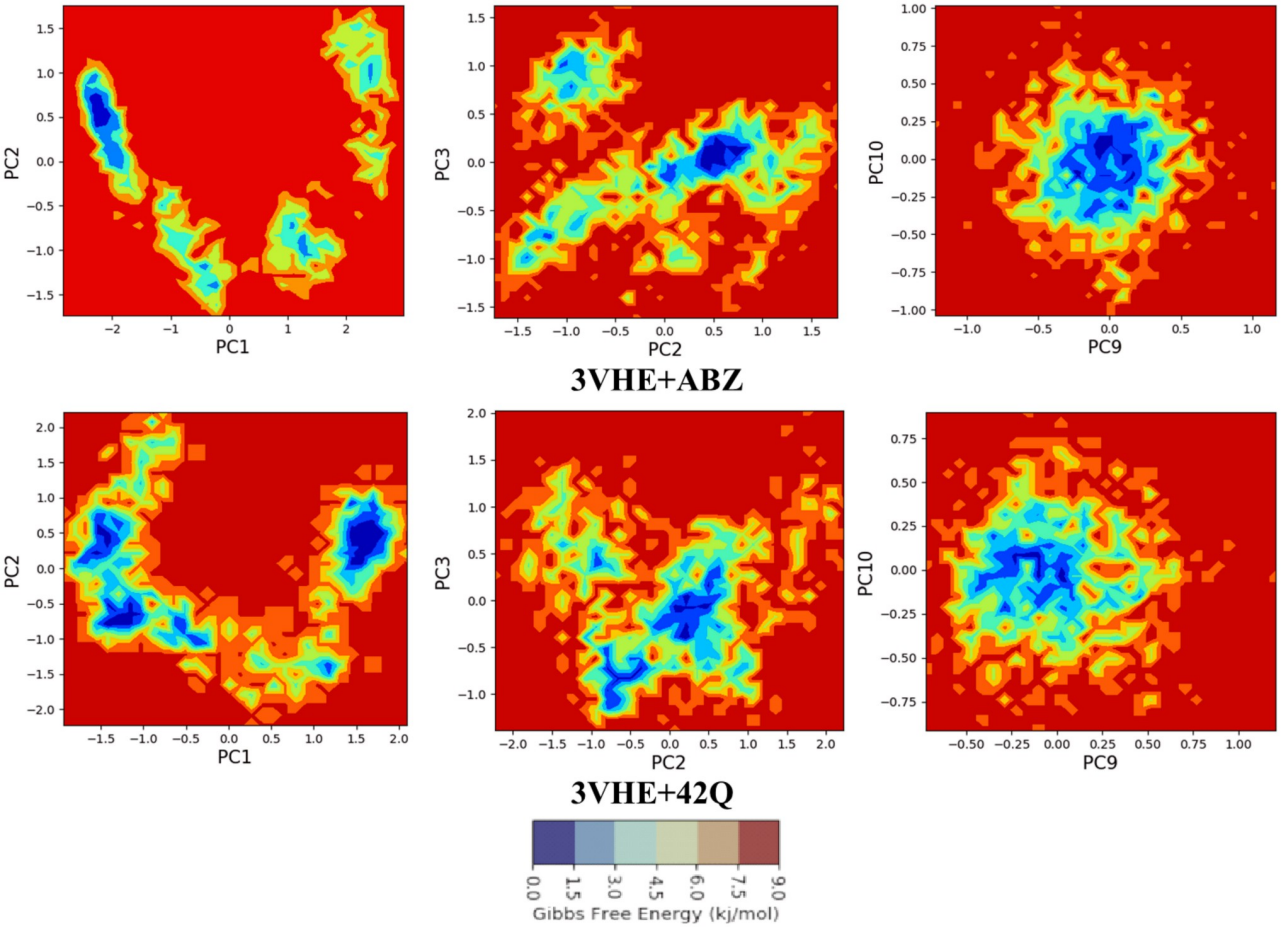
Comparative free energy surface/landscape (FEL) of human VEGFR2 kinase bound with ABZ displaying (a) PC1 and PC2, (b) PC2 and PC3, and (e) PC9 and PC10, for 200 ns simulation trajectories. 42Q with human VEGFR2 kinase domain displaying (d) PC1 and PC2, (e) PC2 and PC3 and (f) PC9 and PC10, for 200 ns simulation trajectories.

Principal component analysis (PCA) of the MD simulation trajectories for ABZ bound to the VEGFR2 kinase domain was analyzed to interpret the randomized, global motion of the atoms of amino acid residues along with free energy. This analysis interprets the more flexible scattered trajectories due to the protein structure’s randomness due to non-correlated global motion. The internal coordinate’s mobility into three-dimensional space in the spatial time of 200 ns was recorded in a covariance matrix. The rational motion of each trajectory is interpreted in the form of orthogonal sets or Eigenvectors. MD simulation trajectory of Cα atoms of ABZ bound to VEGFR2 kinase domain displayed more unordered orientation in PC1 and PC2 modes where, the free energies are not converged localized (Fig 6A, blue, as per scale). Following this, PC2 and PC3 mode displayed better order Eigenvalues for the trajectories with partially ordered free energy surface (Fig 6B). The Eigenvectors displayed a less scattered, more correlated and partially converged free energy surface of the last 50 trajectories (Fig 6B). Most of the trajectories finally settled in PC9 and PC10, where the global motion centred toward the origin of the plot (Fig 6C). Eigenvectors are observed. The global movement of MD trajectories toward a positive correlation Eigen vector indicates that the VEGFR2 kinase domain complex is compact and converged. The high periodic global motion was observed along the PC9 and PC10 planes due to the grouping of trajectories in a single free energy surface at the centre of the PCA plot toward origin. Cantering of the trajectories in a single cluster indicates the periodic motion of MD trajectories due to stable conformational global motion (Fig 6C, blue, as per scale).

On the other hand, the 42Q bound VEGFR2 kinase domain displayed a similar observation for PCA as found in ABZ bound state. The MD trajectories in PC1 and PC2 modes displayed more disoriented distribution and less free energy converged zones (Fig 6D, blue), while in PC2 and PC3 modes, trajectories started organizing more into partially definite free energies (Fig 6E, blue). Interestingly increment of PC modes up to PC9 and PC10 displayed more ordered trajectories. The last 50 trajectories (yellow dots) in PC9 and PC10 showed ordered orientation toward the origin of the Eigenvalues (Fig 6F, blue). Ordered orientation signifies the ordered global motion of the trajectories that arose due to a stable converged structure and high free energies toward zero. Therefore, at this stage global minimization achieved.

## Conclusion

Albendazole approved by FDA for therapeutic usage as an anthelmintic found to act as VEGFR-2 inhibitor, that represent potential treatment option after investigation. Albendazole binds with conserved residues presents in substrate binding pocket and forms remarkable interactions. Molecular docking and dynamics study are well-established computational techniques that predict drugs’ affinity and stability towards the receptor. The current investigation on Albendazole interaction with VEGFR-2 using molecular docking suggested the achievable interaction and possible inhibition. In addition, molecular dynamics simulation suggested greater stability of VEGFR-2 with Albendazole like a standard cocrystal ligand 42Q with plausible and significant binding energies. The results of this study confirm initial reports however additional investigation necessary to check the efficacy of drug by in vitro and in vivo studies followed by clinical trials against ovarian cancer patients. But publishing this information in public domain may help community to fight with ovarian cancer if any potential investigator proceeds for trials. We recommend that this drug candidate be experimentally tested and used as a starting point for further design of a high efficient drug.

## Supporting information

S1 Graphical abstract (DOCX)

## References

[1] Shabir Saba, and Prabhjot Kaur Gill. "Global scenario on ovarian cancer–Its dynamics, relative survival, treatment, and epidemiology." *Adesh University Journal of Medical Sciences & Research* 2, no. 1 (2020): 17–25.

[2] Richter Mark, and Zhang Hongtao. "Receptor-targeted cancer therapy." *DNA and cell biology* 24, no. 5 (2005): 271–282. doi: 10.1089/dna.2005.24.271 15869404

[3] Aggarwal S. (2010). Targeted cancer therapies. *Nature reviews*. *Drug discovery*, 9(6), 427. doi: 10.1038/nrd3186 20514063

[4] Holmes D. I. R. and Zachary I., “The vascular endothelial growth factor (VEGF) family: angiogenic factors in health and disease,” *Genome Biol*., vol. 6, no. 2, p. 209, 2005. doi: 10.1186/gb-2005-6-2-209 15693956 PMC551528

[5] Mahdy H. A. et al., “Design, synthesis, molecular modeling, in vivo studies and anticancer evaluation of quinazolin-4(3H)-one derivatives as potential VEGFR-2 inhibitors and apoptosis inducers,” *Bioorg*. *Chem*., vol. 94, p. 103422, 2020. doi: 10.1016/j.bioorg.2019.103422 31812261

[6] Spannuth W. A., Nick A. M., Jennings N. B., Armaiz‐Pena G. N., Mangala L. S., Danes C. G., et al (2009). Functional significance of VEGFR‐2 on ovarian cancer cells. *International journal of cancer*, 124(5), 1045–1053. doi: 10.1002/ijc.24028 19058181 PMC2668132

[7] Sun S., Schiller J. H., and Gazdar A. F., “Lung cancer in never smokers—a different disease,” *Nat*. *Rev*. *Cancer*, vol. 7, no. 10, pp. 778–790, 2007. doi: 10.1038/nrc2190 17882278

[8] El-Helby A.-G. A., Sakr H., Eissa I. H., Abulkhair H., Al-Karmalawy A. A., and El-Adl K., “Design, synthesis, molecular docking, and anticancer activity of benzoxazole derivatives as VEGFR-2 inhibitors,” *Arch*. *Pharm*. *(Weinheim)*., vol. 352, no. 10, p. 1900113, Oct. 2019. doi: 10.1002/ardp.201900113 31448458

[9] Abdullaziz M. A. et al., “Design, synthesis, molecular docking and cytotoxic evaluation of novel 2-furybenzimidazoles as VEGFR-2 inhibitors,” *Eur*. *J*. *Med*. *Chem*., vol. 136, pp. 315–329, 2017. doi: 10.1016/j.ejmech.2017.04.068 28505536

[10] Dogra N., Kumar A., and Mukhopadhyay T., “Fenbendazole acts as a moderate microtubule destabilizing agent and causes cancer cell death by modulating multiple cellular pathways,” *Sci*. *Rep*., vol. 8, no. 1, p. 11926, 2018. doi: 10.1038/s41598-018-30158-6 30093705 PMC6085345

[11] Pantziarka, P., Meheus, L., Rombauts, K., Vandeborne, L., & Bouche, G. (2020). Drug repurposing for cancer therapy—an introduction. In *Drug repurposing in cancer therapy* (pp. 1–14). Academic Press.

[12] Khattab M. and Al-Karmalawy A. A., “Computational repurposing of benzimidazole anthelmintic drugs as potential colchicine binding site inhibitors,” *Future Med*. *Chem*., vol. 13, no. 19, pp. 1623–1638, Sep. 2021. doi: 10.4155/fmc-2020-0273 34505541

[13] Yadav S., Narasimhan B., and kaur H., “Perspectives of Benzimidazole Derivatives as Anticancer Agents in the New Era,” *Anti-Cancer Agents in Medicinal Chemistry*, vol. 16, no. 11. pp. 1403–1425, 2016. doi: 10.2174/1871520616666151103113412 26526461

[14] Doudican N., Rodriguez A., Osman I., and Orlow S. J., “Mebendazole induces apoptosis via Bcl-2 inactivation in chemoresistant melanoma cells.,” *Mol*. *Cancer Res*., vol. 6, no. 8, pp. 1308–1315, Aug. 2008. doi: 10.1158/1541-7786.MCR-07-2159 18667591

[15] Ghasemi F., Black M., Vizeacoumar F., Pinto N., Ruicci K. M., Le C. C. S. H., et al. (2017). Repurposing Albendazole: new potential as a chemotherapeutic agent with preferential activity against HPV-negative head and neck squamous cell cancer. *Oncotarget*, 8(42), 71512. doi: 10.18632/oncotarget.17292 29069723 PMC5641066

[16] Chu S. W. L., Badar S., Morris D. L., and Pourgholami M. H., “Potent inhibition of tubulin polymerisation and proliferation of paclitaxel-resistant 1A9PTX22 human ovarian cancer cells by albendazole.,” *Anticancer Res*., vol. 29, no. 10, pp. 3791–3796, Oct. 2009. 19846910

[17] Movahedi F., Li L., Gu W., & Xu Z. P. (2017). Nanoformulations of albendazole as effective anticancer and antiparasite agents. *Nanomedicine*, 12(20), 2555–2574. doi: 10.2217/nnm-2017-0102 28954575

[18] Oguro Y., Miyamoto N., Okada K., Takagi T., and Iwata H., “Bioorganic & Medicinal Chemistry Design, synthesis, and evaluation of 5-methyl-4-phenoxy-5 H -pyrrolo- [3, 2- d] pyrimidine derivatives: Novel VEGFR2 kinase inhibitors binding to inactive kinase conformation,” *Bioorg*. *Med*. *Chem*., vol. 18, no. 20, pp. 7260–7273, 2010. doi: 10.1016/j.bmc.2010.08.017 20833055

[19] O’Boyle N. M., Banck M., James C. A., Morley C., Vandermeersch T., and Hutchison G. R., “Open Babel: An open chemical toolbox,” *J*. *Cheminform*., vol. 3, no. 1, p. 33, 2011. doi: 10.1186/1758-2946-3-33 21982300 PMC3198950

[20] Lee K. et al., “Pharmacophore modeling and virtual screening studies for new VEGFR-2 kinase inhibitors,” *Eur*. *J*. *Med*. *Chem*., vol. 45, no. 11, pp. 5420–5427, 2010. doi: 10.1016/j.ejmech.2010.09.002 20869793

[21] Filimonov D. A. et al., “Prediction of the Biological Activity Spectra of Organic Compounds Using the Pass Online Web Resource,” *Chem*. *Heterocycl*. *Compd*., vol. 50, no. 3, pp. 444–457, 2014. doi: 10.1007/s10593-014-1496-1

[22] K. J. Bowers *et al*., “Scalable Algorithms for Molecular Dynamics Simulations on Commodity Clusters,” in *SC ‘06*: *Proceedings of the 2006 ACM/IEEE Conference on Supercomputing*, 2006, p. 43.

[23] E. Chow *et al*., “Desmond Performance on a Cluster of Multicore Processors,” *Simulation*, no. July, pp. 1–14, 2008, [Online]. https://www.deshawresearch.com/publications/DesmondPerformanceonaClusterofMulticoreProcessors.pdf.

[24] Shivakumar D., Williams J., Wu Y., Damm W., Shelley J., and Sherman W., “Prediction of Absolute Solvation Free Energies using Molecular Dynamics Free Energy Perturbation and the OPLS Force Field,” *J*. *Chem*. *Theory Comput*., vol. 6, no. 5, pp. 1509–1519, May 2010. doi: 10.1021/ct900587b 26615687

[25] Jorgensen W. L., Chandrasekhar J., Madura J. D., Impey R. W., and Klein M. L., “Comparison of simple potential functions for simulating liquid water,” *J*. *Chem*. *Phys*., vol. 79, no. 2, pp. 926–935, Jul. 1983. doi: 10.1063/1.445869

[26] Martyna G. J., Tobias D. J., and Klein M. L., “Constant pressure molecular dynamics algorithms,” *J*. *Chem*. *Phys*., vol. 101, no. 5, pp. 4177–4189, Sep. 1994. doi: 10.1063/1.467468

[27] Martyna G. J., Klein M. L., and Tuckerman M., “Nosé–Hoover chains: The canonical ensemble via continuous dynamics,” *J*. *Chem*. *Phys*., vol. 97, no. 4, pp. 2635–2643, Aug. 1992. doi: 10.1063/1.463940

[28] Toukmaji A. Y. and Board J. A., “Ewald summation techniques in perspective: a survey,” *Comput*. *Phys*. *Commun*., vol. 95, no. 2, pp. 73–92, 1996. doi: 10.1016/0010-4655(96)00016-1

[29] Kagami L. P., das Neves G. M., Timmers L. F. S. M., Caceres R. A., and Eifler-Lima V. L., “Geo-Measures: A PyMOL plugin for protein structure ensembles analysis,” *Comput*. *Biol*. *Chem*., vol. 87, p. 107322, 2020. doi: 10.1016/j.compbiolchem.2020.107322 32604028

