# Supplementary figures and images for: Albendazole repurposing on VEGFR-2 for possible anticancer application: In-silico analysis
Source: PLoS One. 2023 Aug 16;18(8):e0287198. doi: 10.1371/journal.pone.0287198 (PMC10431642; doi:10.1371/journal.pone.0287198)

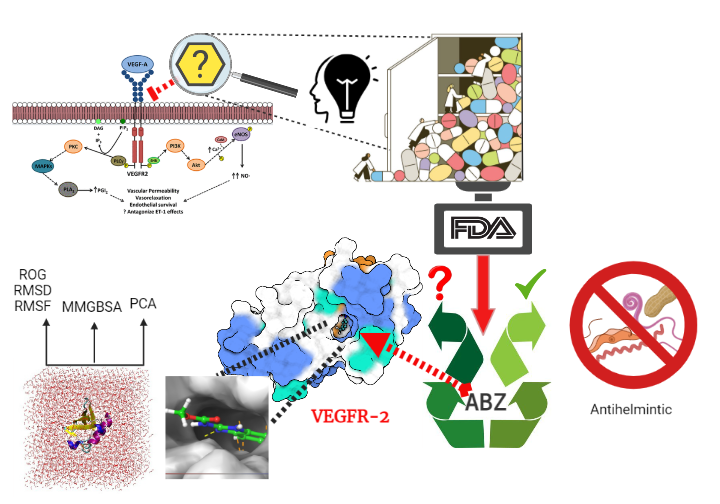

Supplement: S1 Graphical abstract — (DOCX) [file pone.0287198.s001.docx]
